# The effect of team cohesion on mental toughness: a mediation and moderation mixed study based on COR theory

**DOI:** 10.3389/fpsyg.2025.1655398

**Published:** 2025-12-03

**Authors:** Yiguo Xu, Wenhao Tian

**Affiliations:** Dankook University, Yongin, Republic of Korea

**Keywords:** team cohesion, social support, self-identity, mental toughness, athletes

## Abstract

**Objective:**

To investigate the mechanisms through which team cohesion, social support, and self-identity influence athletes’ mental toughness, grounded in Conservation of Resources (COR) theory.

**Methods:**

A total of 523 valid questionnaires were collected from athletes aged 16–35 years using the Team Cohesion Scale, Social Support Scale, Self-Identity Scale, and Mental Toughness Scale. The collected data was validated through correlation analysis, regression analysis, and other methods.

**Results:**

(1) Team cohesion has a significant positive effect on athletes’ mental toughness. (2) Team cohesion influences mental toughness both directly and indirectly through the mediating roles of social support and self-identity, encompassing three mediation pathways: social support alone, self-identity alone, and a chained mediation through both social support and self-identity.

**Conclusion:**

Team cohesion not only directly enhances athletes’ mental toughness but also indirectly influences it through the independent and chained mediating effects of social support and self-identity. Future interventions could focus on enhancing team cohesion to increase athletes’ social support and strengthen their self-identity, thereby improving mental toughness.

## 1 Introduction

The research topic of psychological resilience first gained attention from American psychologists in the 1970s. Initially, research on psychological resilience focused primarily on children. By the 1990s, studies on psychological resilience advanced further, beginning to examine the influence of external environments, social support, and other factors on psychological resilience. Psychological resilience is considered a crucial psychological trait for achieving outstanding performance. To excel in competitions and training, athletes require a high level of psychological resilience (Chen et al., 2025). Positive psychologist Fred Luthans defines psychological resilience as “the potential individuals can develop to recover quickly from failure, adversity, and increasing responsibilities” (Tang and Wang, 2024). The psychological resilience development model posits that resilience is a positive psychological quality acquired through an individual’s interaction with adverse environments (Han Zhongxu, 2025). It is now recognized as a core trait that enables athletes to achieve outstanding performance (Chrétien et al., 2024), and maintaining a high level of mental toughness is essential during competition (Cowden, 2017). Athletes with greater mental toughness are able to sustain high levels of competitive performance despite encountering setbacks (Powell and Myers, 2017), whereas those with lower levels of mental toughness tend to exhibit maladaptive responses, such as excessive anxiety, self-doubt, lack of confidence, and emotional outbursts (Stewart et al., 2025). In this context, mental toughness exerts a direct influence on both the competitive performance and psychological well-being of athletes. Particularly as competitive sports environments become increasingly high-pressure, how to effectively enhance athletes’ psychological resilience has emerged as a key research topic in sports psychology. However, while existing studies have largely focused on the outcome effects of psychological resilience (Galli and Gonzalez, 2015), few have explored its developmental mechanisms from the perspective of group factors or team characteristics, leaving room for further investigation. Therefore, identifying the factors that influence mental toughness and uncovering its underlying mechanisms hold significant practical value. Developing effective strategies to enhance mental toughness can enable athletes to respond constructively to challenges arising from competition and daily life, thereby promoting both physical and mental health. Among the various influencing factors, team cohesion fosters a strong sense of belonging and identity among athletes. By reinforcing social support, shared goals, and emotional bonds, team cohesion helps athletes maintain high levels of mental toughness in the face of adversity (Carron et al., 2002). Previous studies have categorized the development of mental toughness into internal and external factors. Internal factors primarily include cognitive styles and personality traits, while external factors encompass environmental stimuli and parenting styles (Broll et al., 2025). Gucciardi (2012) suggested that mental toughness is, to some extent, associated with self-determination theory. Butt et al. (2010) proposed that mental toughness comprises personality traits such as focus, self-confidence, positive thinking, and emotional regulation. After reading many studies, the author found that researchers have looked at the link between team cohesion and psychological resilience, but they have not clearly explained how the two work together. Research based on Chinese competitive sports is also very limited. So, this study focuses on athletes to explore how team cohesion helps improve psychological resilience. It tries to fill the gap in data and theory and offers both a theoretical base and practical ways to strengthen athletes’ psychological resilience.

## 2 Theoretical foundations

### 2.1 Conservation of resources theory

The fundamental principle of Conservation of Resources Theory (COR) is that individuals or teams endeavor to preserve, protect, and build resources they deem valuable, with the loss of these resources constituting a significant threat (Liao et al., 2022). Broadly speaking, a resource is defined as anything that an individual values. Hobfoll categorizes resources into material resources (e.g., survival environment, work), conditioned resources (e.g., social support, social relationships), personality traits (e.g., self-efficacy, self-esteem), and energetic resources (e.g., time, money). These resources not only meet an individual’s needs but also assist in self-identification and social orientation (Hobfoll, 1989). The core idea of COR theory is that individuals with more resources are less susceptible to threats posed by resource loss and are more capable of acquiring additional resources, and vice versa. This introduces two spirals of resource dynamics: the loss spiral and the acquisition spiral. The loss spiral occurs when individuals lacking resources are vulnerable to the stress of resource loss, and the presence of this stress often exacerbates the loss of resources, creating a cycle that accelerates resource depletion. Conversely, the acquisition spiral refers to individuals with ample resources or social support, who are not only more capable of acquiring additional resources but also experience a cumulative increase in resources (Liu et al., 2025). It is important to note that the rate of resource acquisition is much slower than the rate of resource loss, making individuals with limited resources more prone to falling into the loss spiral.

In summary, Conservation of Resources (COR) theory bears significant relevance to this study. First, the core idea of COR theory is “resources.” These include material, conditional, personality trait, and energy resources. The meaning of “resources” is very broad. In this study, team cohesion and social support are seen as conditional resources. Self-identity is seen as a personality trait resource. The speed at which people gain and lose these two kinds of resources together affects their mental toughness (Holmgreen et al., 2017). Secondly, COR theory is not a static framework merely describing the relationship between variables, but rather a dynamic theory that emphasizes the “process,” particularly the spirals of resource acquisition and resource loss (Farkash et al., 2022). Individuals with more initial resources (e.g., high cohesion, team rapport, high sense of security) facilitate the acquisition of additional resources (e.g., support and help from teammates), thereby enabling the individual to develop more resources (e.g., mental toughness); conversely, the lack of such resources leads to diminished resource acquisition and greater vulnerability to resource loss (Wilson and Young, 2023). Finally, COR theory is intrinsically relevant to stressful situations. Although this study did not directly explore the mechanisms of stress, it has been clarified in the previous section that the core concept of mental toughness involves an individual’s effective adaptation to stressful situations such as frustration and adversity. Therefore, using COR theory as a framework to explain the influence of team cohesion, social support, and self-identity on mental toughness provides logical consistency and explanatory power.

### 2.2 Mental toughness theory

In psychology, mental toughness is a positive quality that helps people deal with stress and hardship. It has been studied for a long time. Research on “resilience” started in the United States. When Chinese scholars brought this idea into China, they used different translations. Scholars in Taiwan often use “resilience,” scholars in Hong Kong often say “adversity resilience,” and scholars in mainland China mostly use “mental toughness.” There is still no single clear definition of mental toughness. After looking at many studies, most scholars accept one main view: mental toughness means positive adjustment when facing difficulty. There are two main conditions: (1) there is adversity or danger, and (2) the person can still adapt well even though these problems strongly affect their development. These two parts form the core of mental toughness. From this view, the main idea of mental toughness includes two parts: “adversity” and “adaptation.” These match risk factors and protective factors. Risk factors are biological, environmental, or psychosocial things that make negative outcomes more likely. Protective factors are things that reduce or block the effects of risk factors and lower the chances of problem behaviors. The ideas above have led to different theories. Mandleco (2000) suggested a systemic model that sees a person as a whole made up of inside and outside factors. Inside factors include psychological traits. Outside factors include social, family, and other environmental parts. This model stresses that the system works as a whole and sees psychological resilience as the result of many factors together. When facing hardship, people improve their psychological resilience by using both outside and inside resources. In this study, team cohesion and social support can act as outside factors that help build mental toughness. Self-identity can act as an inside factor that supports it. So, using mental toughness theory in this research is suitable.

## 3 Research hypotheses

### 3.1 The relationship between team cohesion and mental toughness

The concept of team cohesion as a dynamically evolving construct has undergone a paradigm shift from a static unidimensional view to a systemic multidimensional view (Liao, 2020). Early studies primarily viewed team cohesion as a unidimensional structure but struggled to explain the mechanisms of team cohesion in complex situations (Evans et al., 1986). Subsequent research has explored these mechanisms from a multidimensional perspective, which has become more widely accepted in the academic community (Gross and Martin, 1952). The multidimensional structure divides team cohesion into task cohesion and relational cohesion. Task cohesion refers to the commitment to or attraction of a group task or goal, while relational cohesion refers to the attraction between group members or their affinity toward the group (Rios and Mackey, 2020). Task cohesion enhances group members’ effort on the task, while relational cohesion enhances emotional communication and effectively synergizes team efforts. According to existing research, team cohesion fosters interpersonal attraction among team members and motivates them to work toward a common goal. In this context, highly cohesive teams foster strong interpersonal relationships and a sense of security, allowing individuals to feel understanding and support from teammates when facing setbacks (Muñoz et al., 2023). This, in turn, can significantly enhance athletes’ mental toughness (Gu and Xue, 2022). In the context of COR theory, team cohesion can be regarded as an important “conditional resource.” A highly cohesive team provides a stable, harmonious, and supportive environment, where individuals benefit from richer resource reserves and enhanced ability to resist resource loss. Consequently, individuals in such environments can maintain stability under stress, thus exhibiting higher psychological resilience. Moreover, more cohesive teams are more likely to share common goals and strong collective motivation. When individuals work toward these shared goals, their sense of mission and responsibility is further reinforced (Zamecnik et al., 2024). This shared motivation builds athletes’ inner drive and helps them stay strong when they face setbacks. A study on police academy students showed that team cohesion has a clear positive effect on individual psychological resilience (Wang et al., 2019). Research on cohesion has moved from one-dimensional to multi-dimensional views. Cohesion mainly gives people a stable and supportive environment for growth. This study looks at how individual psychological resilience develops, and cohesion is an important factor in this process. The research hypothesis is: H1: Team cohesion has a clear positive relationship with psychological resilience.

### 3.2 The role of social support in the relationship between team cohesion and mental toughness

Social support refers to the emotional or material help provided to an individual by those closely connected to them (Liu et al., 2025). With respect to content, social support can be categorized into instrumental, emotional, informational, and peer support (Acoba, 2024). With respect to its nature, social support can be classified as either actual or perceived support (Gutiérrez-Sánchez et al., 2024). The antecedent variables of social support include various factors such as individual characteristics (Liao et al., 2025), interpersonal relationships (Shin and Park, 2022), and the broader social environment (Choi et al., 2024). Athletes often train in a closed and insular environment that fosters a unique group ecology. As a result, they experience greater pressure from both competition and daily life compared to the general population, necessitating greater support from coaches and teammates. Teams with high cohesion tend to reinforce supportive behaviors among members through emotional resonance (Aldrich and Meyer, 2015). According to team efficacy theory, cohesion enhances communication and trust among members, increasing individuals’ willingness to actively seek help and support when facing challenges, thereby creating a positive “cohesion–support” cycle (Kozlowski and Ilgen, 2006). Empirical studies have demonstrated that team cohesion significantly influences athletes’ daily lives and performance, serving as a key source of social support (Cao and Chi, 2016).

In recent years, social support has been widely recognized as a critical external resource influencing mental toughness, with many psychologists examining its antecedents through the lens of risk and protective factors (Pei, 2024). Social support buffers the impact of life stressors and thus functions as a protective factor in the development of mental toughness (Yıldırım et al., 2025). Yao Shang et al. (2021) emphasized that social support plays a crucial role in the development of mental toughness among athletes, particularly following competition losses and performance-related setbacks, as it not only enhances athletes’ problem-solving capabilities but also reduces their maladaptive appraisal of adversity. Empirical evidence further confirms that social support significantly and positively predicts mental toughness (Kapikiran and Bulbuloglu, 2024). Furthermore, neuromechanistic research suggests that individuals with high levels of social support exhibit enhanced functional connectivity between the prefrontal cortex and the ventral striatum under stress, a change in neuroplasticity that is closely associated with adaptive emotion regulation strategies (Koizumi et al., 2016). Within the framework of COR theory, a highly cohesive team fosters favorable conditions for the emergence of social support, as mutual trust and reciprocal assistance are more likely to occur among members of cohesive teams. This, in turn, enables individuals to better cope with challenges, prevent resource depletion, and ultimately strengthen mental toughness. In short, there is little research on how social support works between team cohesion and mental toughness. Most studies only look at simple links between two variables. So, this study will explore how team cohesion affects mental toughness through an internal process. The research hypothesis is H2: Social support acts as a mediator in the positive effect of team cohesion on mental toughness.

### 3.3 The role of self-identity in the relationship between team cohesion and mental toughness

Self-identity refers to the process by which individuals, through interactions with others and engagement in social practices, develop self-reflection via an internal reference system, ultimately leading to the gradual development of behavioral and cognitive consistency (Giddens, 2023). Anthony Giddens conceptualizes self-identity from a social-semiotic perspective, proposing that the “main self,” “object self,” and perspectives of others collectively constitute the discursive features of self-identity (Jia, 2003). Within this framework, the “guest self” reflects the influence of social recognition in the formation of self-consciousness. Several Chinese scholars emphasize the significant role of the social environment in shaping self-identity, noting that internal processes such as empathy and emotional experience are socially mediated (Yao and Luo, 2011). Applying these theoretical perspectives to the current study, team cohesion—as a form of external environmental stimulation—can influence the development of team members’ self-identity. Specifically, teams characterized by high cohesion offer greater social support and collective motivation. In such contexts, team members develop a strong sense of belonging and mutual identification, which in turn reinforces their self-identity through perceived emotional connections and interactions. Cohesion has therefore been identified as a critical factor in self-identity development, with empirical studies showing that team cohesion can reduce anxiety and enhance athletes’ self-identity (Heuzé et al., 2006).

People with a strong sense of self-identity often feel positive psychological effects. Self-identity has a clear positive link with positive emotions and a clear negative link with negative emotions (Ryan and Deci, 2000). In Mandleco’s mental toughness theory, self-identity is seen as an inside factor that helps build mental toughness. So, it makes sense to say there is a close link between self-identity and mental toughness. Stable self-identity also helps people see setbacks as “chances to grow” instead of “life threats” (Vignoles et al., 2016). Past studies have also shown that self-identity has a clear positive effect on mental toughness (Jin, 2022). In short, this study expects that teams with higher cohesion can give individuals more help and support. In this kind of environment, people’s sense of identity will keep growing, and they will handle setbacks in competitions better. Accordingly, Hypothesis H3 is proposed: self-identity mediates the positive relationship between team cohesion and mental toughness.

### 3.4 Chain mediation model

Although the positive effect of team cohesion on mental toughness has been well documented, the internal mechanisms underlying this relationship remain insufficiently explored—often referred to as the “black box” problem in existing research. To address this, the present study proposes a chain mediation model in which social support and self-identity function as sequential mediators. Attachment theory suggests that emotional support in early life plays a critical role in forming an “internal working model” that strengthens self-concept (Bowlby, 1969). In adulthood, social support—particularly from teams or peers—continues to shape self-perceptions through processes such as “mirroring,” whereby individuals internalize supportive feedback from others. Further, Ryan (2017) and colleagues posit that self-identity is grounded in the fulfillment of basic psychological needs. Strong social support contributes to this by enhancing individuals’ sense of autonomy, competence, and relatedness—core components of self-determination theory. COR theory says that people who already have many resources can gain more and also get more value from what they gain. When people feel a high level of social support in a team, this good experience strongly shapes their self-identity (Liu et al., 2025). In supportive settings, people are more likely to show their true selves and explore their abilities, which helps build self-identity. Mental toughness theory also says that a person’s level of resilience comes from both inside and outside factors. Cohesion and social support are outside factors. Self-identity is an inside factor. Based on the step-by-step mediation in this study, outside environmental resources (team cohesion) help people get conditional resources (social support). After people gain social resources, they are more likely to build personality trait resources (self-identity). In the end, having many resources improves their ability to adapt (mental toughness).

**Figure 1:**
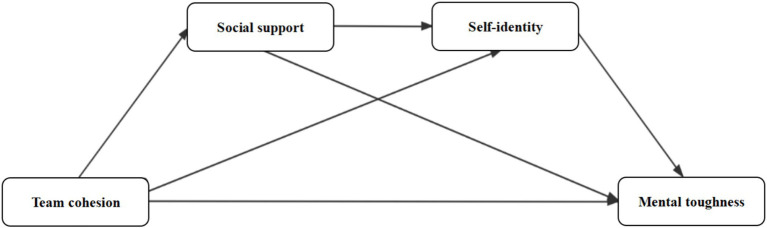
Conceptual model proposed in this study.

Integrating research hypotheses H1 through H3, this study further proposes a sequential mediation path: team cohesion → social support → self-identity → psychological resilience. That is, individuals embedded in highly cohesive teams are more likely to perceive strong social support; this support strengthens their self-identity, which in turn enhances their ability to adapt effectively to challenges and adversity. This internal mechanism reflects a resource enrichment process consistent with COR theory. Accordingly, Hypothesis H4 is proposed: Team cohesion positively influences mental toughness through the chain-mediated effects of social support and self-identity (see Figure 1).

## 4 Method

### 4.1 Research subjects

This study employed a stratified random sampling method to select professional athletes at the provincial level and above, collegiate athletes, and professional club athletes from various regions of China, including East China, North China, South China, and West China. The aim was to examine the mechanisms through which team cohesion influences athletes’ mental toughness. Participants ranged in age from 16 to 35 years (*M* = 25.4, SD = 5.8) and represented multiple sports disciplines, including team sports (basketball, soccer, volleyball, etc., accounting for 51.1%) and individual sports (swimming relays, track and field 4 × 100, team gymnastics, etc., accounting for 48.9%), to ensure the applicability of findings across different athletic categories. A total of 600 questionnaires were distributed, with 523 valid responses received after excluding invalid questionnaires (e.g., incomplete answers, patterned responses, or obvious contradictions), yielding an effective recovery rate of 87.2%. Among the valid responses, 47.6% were from male athletes and 52.4% from female athletes. In terms of competitive level, 37.3% were national-level and above athletes, 27.5% were national-level 2 athletes, and 35.2% were unclassified athletes. The mean number of years of team training was 4.5 years (SD = 2.2), with 67.7% of athletes having three or more years of team experience. All participants volunteered for the study and provided informed consent, acknowledging their understanding of the study’s purpose, confidentiality principles, and data anonymity protocols. The study was approved by the Ethics Committee of Zhengzhou University (ZZUIRB2024-0833), in compliance with the Code of Ethics for Research in Sport Psychology. The sample size met the criteria for Structural Equation Modeling (SEM) analysis (Kline, 2023), and a statistical power analysis (1-*β* > 0.90) was conducted using G*Power 3.1 to ensure the adequacy of the sample size for testing the chain-mediated effects. Furthermore, to control for common method bias (CMV), Harman (1976) one-way test was applied, ensuring the validity and reliability of the data.

### 4.2 Research instruments

#### 4.2.1 Team cohesion scale

The Group Environment Questionnaire (GEQ), revised by Ma Hongyu (2004), was employed in this study to assess team cohesion. The questionnaire comprises 15 items, including 13 positively worded and 2 negatively worded items, measuring individuals’ commitment to two dimensions: group task cohesion and group social cohesion. Responses were recorded on a 7-point Likert scale (1 = strongly disagree, 7 = strongly agree). For positively worded items (e.g., “Our team is united in achieving the goal”), higher scores indicate stronger team cohesion; conversely, for negatively worded items (e.g., “Members of our team prefer going out alone to doing activities together”), lower scores denote higher team cohesion. The scale is well-suited to the Chinese cultural context and demonstrates strong reliability and validity in this study: the internal consistency coefficient (Cronbach’s *α*) is 0.911. Confirmatory factor analysis indicates good model fit (χ^2^/df = 1.670, CFI = 0.979, TLI = 0.975, RMSEA = 0.036, SRMR = 0.029), confirming the scale’s effectiveness in measuring team cohesion among athletes.

#### 4.2.2 Social support scale

Social support was measured using the Social Support Scale developed by Vaux et al. (1986) and revised by Xin et al. (2007). The scale consists of 20 question items on a 5-point Likert scale (1 = never, 5 = always), with a total score range of 20–100, with higher scores indicating higher levels of perceived social support. The scale covers three dimensions: emotional support, instrumental support, and evaluative support. In this study, the Cronbach’s alpha coefficient for the total scale was 0.934, and the alpha coefficients for the dimensions ranged from 0.862–0.891; the results of the validated factor analysis showed a good fit of the model (χ^2^/df = 1.973, CFI = 0.962, TLI = 0.957, RMSEA = 0.043, SRMR = 0.061). These psychometric indicators suggest that the scale is effective in assessing athletes’ perceived level of social support.

#### 4.2.3 Self-identity scale

Self-identity was measured using the Self-Identity Scale developed by Ochse and Plug (1986). The scale consists of 19 items and is rated on a 4-point Likert scale (1 = not at all applicable, 4 = fully applicable), yielding a total score between 19 and 76; higher scores indicate a stronger sense of self-identity. The content of the scale reflects individuals’ cognitive evaluations of self-worth, social role perception, and future orientation. In this study, the scale demonstrated strong psychometric properties: the Cronbach’s *α* coefficient was 0.924, and confirmatory factor analysis showed good model fit (χ^2^/df = 1.754, CFI = 0.972, TLI = 0.969, RMSEA = 0.038, SRMR = 0.037). These results suggest that the scale is effective in assessing self-identity among athletes.

#### 4.2.4 Mental toughness scale

Mental toughness was measured using the Chinese version of the Connor-Davidson Resilience Scale (CD-RISC), revised by Yu et al. (2011). The scale comprises 25 items divided into three dimensions: strength (10 items), toughness (8 items), and optimism (7 items). It is rated on a 5-point Likert scale (1 = not at all true, 5 = completely true), resulting in a total score between 25 and 125; higher scores reflect greater mental toughness. The scale has demonstrated robust psychometric properties in Chinese athlete populations. In this study, the Cronbach’s α for the overall scale was 0.937, with dimension-specific α values ranging from 0.856 to 0.902. Results of confirmatory factor analysis indicated good model fit (χ^2^/df = 1.775, CFI = 0.956, TLI = 0.952, RMSEA = 0.039, SRMR = 0.066), confirming that the scale effectively assesses mental toughness among athletes.

### 4.3 Data collection and analysis

This study used an online questionnaire on the “QuestionStar” platform to survey professional athletes in East, North, South, and West China. A total of 600 questionnaires were sent out. To keep the data reliable, we removed questionnaires that were incomplete, had patterned answers, or had clear contradictions. In the end, 523 valid questionnaires were collected, giving an effective response rate of 87.2%. During the questionnaire design, all questions were anonymous to get honest answers. The first paragraph included instructions and notes to explain the research purpose and how to complete the questionnaire. We used SPSS 26.0 to run descriptive statistics, correlation analysis, regression analysis, and tests for common method bias. The moderated mediation model was tested using the SPSS macro Process Model 6.

## 5 Analysis of results

### 5.1 Common method bias test

The analysis included 79 items, from which 13 factors were extracted. The first factor explained 13.435% of the total variance, well below the commonly accepted threshold of 40% (Tang and Wen, 2011), suggesting that common method bias is unlikely to pose a significant threat to the validity of the study’s results. The cumulative explained variance reached 65.923%, surpassing the commonly accepted threshold of 60%, indicating that the 13 extracted factors accounted well for the variance in the data set.

### 5.2 Correlation analysis

Table 1 presents the results of descriptive statistics and correlation analysis for each study variable. Among the variables, team cohesion had the highest mean score (*M* = 4.043, SD = 1.346), followed by mental toughness (*M* = 3.026, SD = 0.951), social support (*M* = 3.010, SD = 0.962), and self-identity, which had the lowest mean (*M* = 2.548, SD = 0.854). Correlation analysis revealed that team cohesion was significantly positively associated with mental toughness (*r* = 0.330, *p* < 0.01), social support (*r* = 0.283, *p* < 0.01), and self-identity (*r* = 0.230, *p* < 0.01). Furthermore, mental toughness was significantly positively correlated with both social support (*r* = 0.238, *p* < 0.01) and self-identity (*r* = 0.283, *p* < 0.01), while social support was also positively correlated with self-identity (*r* = 0.240, *p* < 0.01). These results provide preliminary support for subsequent mediation effect analyses.

**Table 1:** Descriptive statistics and correlation analysis of study variables.

Variable	*M*	SD	Team cohesion	Mental toughness	Social support	Self-identity
Team cohesion	4.043	1.346	1			
Mental toughness	3.026	0.951	0.330**	1		
Social support	3.010	0.962	0.283**	0.238**	1	
Self-identity	2.548	0.854	0.230**	0.283**	0.240**	1

### 5.3 Chained mediation model

A moderated mediation model was tested using the PROCESS macro in SPSS, specifically employing Model 6, to examine the sequential mediating roles of social support and self-identity in the relationship between team cohesion and mental toughness, as follows:

Table 2 presents the results of examining the sequential mediating roles of social support and self-identity in the relationship between team cohesion and mental toughness using the PROCESS macro (Model 6) developed by Hayes (2013). Regression analyses revealed that team cohesion significantly predicted mental toughness (*β* = 0.232, *t* = 7.937, *p* < 0.001), social support (*β* = 0.232, *t* = 7.075, *p* < 0.001), and self-identity (*β* = 0.122, *t* = 4.427, *p* < 0.001). After controlling for demographic variables such as gender and age, social support was a significant positive predictor of both self-identity (*β* = 0.171, *t* = 4.451, *p* < 0.001) and mental toughness (*β* = 0.123, *t* = 2.913, *p* < 0.01). Additionally, self-identity significantly predicted mental toughness (*β* = 0.199, *t* = 4.193, *p* < 0.001). Notably, athlete rank emerged as a significant negative predictor of mental toughness (*β* = −0.109, *t* = −2.439, *p* < 0.05). All regression models were statistically significant (*F* values ranging from 7.590 to 13.501, *p* < 0.001), with explained variances ranging from 8.1 to 17.4%. These findings indicate that social support and self-identity serve as sequential mediators between team cohesion and mental toughness, thereby supporting the research hypothesis.

**Table 2:** Results of chain mediation model testing between team cohesion and mental toughness.

Variable	Mental toughness		Social support		Self-identity		Mental toughness	
	*β*	*t*	*β*	*t*	*β*	*t*	*β*	*t*
Team cohesion	0.232	7.937***	2.282	7.075***	0.122	43427***	0.176	5.832***
Social support					0.171	4.451***	0.123	2.913**
Self-identity							0.199	4.193***
Gender	0.003	0.032	−0.001	−0.009	0.039	0.546	−0.005	−0.067
Age	−0.008	−1.196	−0.003	−0.406	−0.004	−0.716	−0.007	−1.031
Sporting event	0.114	1.445	−0.041	−0.502	−0.017	−0.241	0.124	1.614
Athletic rating	−0.095	−2.061*	0.012	0.242	0.064	1.542	−0.109	−2.439
Team training years	0.003	0.157	0.004	0.238	−0.010	−0.632	0.004	0.238
*R*	0.35		0.285		0.324		0.417	
*R*2	0.122		0.081		0.105		0.174	
*F*	11.982***		7.590***		8.637***		13.501***	

Table 3 presents the results of the bootstrap analysis conducted to assess the significance of the mediation paths between team cohesion and mental toughness. The total effect of team cohesion on mental toughness was significant (effect = 0.232, Boot SE = 0.029, 95% CI [0.175, 0.290]). When controlling for mediators, the direct effect remained significant (effect = 0.176, Boot SE = 0.030, 95% CI [0.117, 0.236]). The total indirect effect through the proposed mediators was also significant (effect = 0.056, Boot SE = 0.019, 95% CI [0.023, 0.096]). Specifically, the indirect effect through social support alone was significant (effect = 0.025, 95% CI [0.002, 0.054]), as was the path through self-identity alone (effect = 0.024, 95% CI [0.005, 0.052]). Moreover, the sequential mediation path via both social support and self-identity was also statistically significant (effect = 0.007, 95% CI [0.001, 0.016]). These results confirm the presence of both individual and chained mediation effects, further supporting the hypothesized model.

**Table 3:** Bootstrap test of the chain mediation model between team cohesion and mental toughness.

Path	Effect value	Boot SE	Boot LLCI	Boot ULCI
Total effect	0.232	0.029	0.175	0.290
Direct effect	0.176	0.030	0.117	0.236
Total indirect effect	0.056	0.019	0.023	0.096
Team cohesion → Social support → Mental toughness	0.025	0.013	0.002	0.054
Team cohesion → Self-identity → Mental toughness	0.024	0.012	0.005	0.052
Team cohesion → Social support → Self-identity → Mental toughness	0.007	0.004	0.001	0.016

## 6 Discussion

### 6.1 Direct effect of team cohesion on mental toughness

The findings of this study indicate that team cohesion significantly and positively predicts psychological resilience, consistent with the research results of Williams et al. (2016) and others. Teams with higher cohesion provide members with stable and reliable emotional connections. Team cohesion encompasses relational cohesion and task cohesion. Relational cohesion fosters an interpersonal atmosphere characterized by shared responsibility, resource sharing, and mutual support, which directly constitutes an individual’s “buffer pool” when facing stress. First, based on COR theory, high team cohesion gives athletes a “psychological resource bank.” Sports competitions have much uncertainty and pressure. Athletes often face possible “loss of resources,” such as losing games or worrying about injuries. In teams with strong cohesion, shared goals and close emotional ties help protect members from losing psychological resources under outside pressure. When people have enough starting resources, like cohesion and social support, they can handle negative outside factors better. They can also avoid a “loss of resources” cycle and may even start a “gain of resources” cycle. Second, based on mental toughness theory, team cohesion is an important “outside resource” that helps build athletes’ positive psychological qualities. In teams with high cohesion, the mental effort needed to ask for help is lower, so people are more willing to seek support and share problems. This helps them get the outside resources they need to deal with stress and directly build mental toughness (Morgan et al., 2013). Finally, team cohesion strengthens mental toughness by reinforcing common goals and collective motivation. Task cohesion refers to members’ identification with team goals and their willingness to collaborate in achieving them. When individuals strongly identify with these goals and perceive mutual responsibility, they interpret personal setbacks within a broader collective framework. This sense of collectivism allows individuals to view challenges as part of fulfilling the team’s commitments which fosters a heightened sense of responsibility and mission, ultimately enabling greater resilience in high-pressure situations, this is consistent with the findings of Shao et al. (2023).

### 6.2 The mediating role of social support

The results of this study suggest that social support partially mediates the relationship between team cohesion and mental toughness. On the one hand, team cohesion provides a “fertile ground” for the emergence of social support; according to Grossman et al. (2022), highly cohesive teams are strongly attracted to one another, maintain positive interpersonal relationships, and share clear common goals, all of which significantly enhance the density and quality of the team’s social support network. In this positive team atmosphere, it is easier to establish a sense of belonging and trust among members, which makes individuals more inclined to proactively offer support (e.g., listening, encouragement) to others. Team members are also more likely to seek and receive effective assistance when needed. In this study, team cohesion had a significant positive predictive effect on social support (*β* = 2.282, *p* < 0.001). This suggests that a more cohesive team can shape a supportive interpersonal environment, facilitating individuals’ access to emotional support or practical help from teammates or coaches. On the other hand, social support serves as the cornerstone for building mental toughness. As a crucial external factor in an individual’s coping with stress and adversity, social support effectively buffers the negative impact of adverse events on athletes. When athletes perceive support, understanding, and encouragement from coaches and teammates, their coping strategies become more diverse. According to mental toughness theory, social support gives people important “protective factors” that help them adapt better when they face losses in competition or problems in training. Specifically, emotional support helps reduce athletes’ anxiety and frustration. Instrumental support gives clear ways to solve problems. Evaluative support helps athletes think about themselves in a positive way. In COR theory, enough social support is seen as an important conditional resource. It helps athletes stop the “loss of resources” cycle and start the “gain of resources” cycle when facing difficulties. This keeps and strengthens their mental toughness. The results of the mediation analysis (effect value: 0.025, 95% CI [0.002, 0.054]) indicate that team cohesion indirectly strengthens individual mental toughness by enhancing social support, thereby affirming the resource transformation chain proposed by COR theory. In essence, higher team cohesion creates favorable conditions for resource acquisition, enabling team members to access the key resource of social support, which in turn strengthens individual mental toughness.

### 6.3 Mediating role of self-identity

The results of this study suggest that self-identity partially mediates the relationship between team cohesion and mental toughness. First, team cohesion, as a resource input, facilitates the development of self-identity. This study found that high levels of team cohesion significantly enhance athletes’ sense of self-identity. From Giddens (2023) sociological semiotics perspective, athletes’ self-identity is not formed in isolation but is shaped through continuous interaction with the external environment via social recognition from others. Teams with high cohesion usually have stronger emotional ties, clearer shared goals, and more frequent communication. In these teams, people are more likely to feel a sense of belonging and acceptance (Moustakas, 2025). Lakhmani et al. (2022) and others also found that cohesion helps increase belonging and group pride. From the view of COR theory, teams with high cohesion are an important “conditional resource” that gives athletes social support, emotional connection, and other resources. Getting these resources lowers the chance of identity problems and helps people build positive and stable self-identities. Athletes feel accepted and valued in the team, which helps them form a more stable self-identity (Pepple and Davies, 2019). Secondly, once established, self-identity functions as a powerful internal psychological resource. According to COR theory, individuals with greater resource reserves are better equipped to manage stress and adversity, as they can defend against potential losses and invest in future gains. High levels of self-identity exert several important effects: (1) Individuals develop a clearer self-concept, allowing them to maintain stability in the face of setbacks and avoid significant resource depletion resulting from negative external evaluations; (2) they are more likely to perceive stressful events as opportunities for growth rather than threats, thereby encouraging the adoption of constructive coping strategies; and (3) self-identity reinforces a sense of purpose and meaning, enhancing intrinsic motivation to remain resilient during adversity (Van der Werff et al., 2013). Thus, self-identity functions as a mediator in the process by which team cohesion influences mental toughness. This mediating mechanism aligns with the core tenets of COR theory—resource acquisition, transformation, and accumulation—and illuminates the ways in which the group environment shapes individual psychological resources. From a practical standpoint, fostering mental toughness not only requires cultivating a highly cohesive team environment, but also necessitates nurturing members’ self-identity. Helping individuals find belonging and personal value within the team ultimately strengthens their ability to cope with stressful situations.

### 6.4 Chain mediating role of social support and self-identity

This study found that team cohesion can affect individuals’ mental toughness through the chain-mediated effects of social support and self-identity. In highly cohesive teams, members interact frequently, maintain strong relationships, and are mutually attracted to one another, thereby fostering trust and a positive interpersonal climate. This environment significantly reduces the psychological cost of seeking external support and facilitates the perception of both instrumental (e.g., information, guidance) and affective support (e.g., encouragement, caring) from teammates and coaches (Morelli et al., 2015). When individuals receive consistent support from the team, particularly emotional support, it not only buffers stress and alleviates negative emotions but, more importantly, conveys the sense of being “accepted” and “valued.” According to social identity theory, recognition from in-group members can effectively satisfy an individual’s need for competence and belonging (Hogg, 2016). When an individual perceives themselves as an important member of a team, it facilitates the internalization of the team’s values and norms, reinforces their identity within the group, and ultimately promotes the development of a clear and stable sense of self. Individuals possessing stable and strong self-identity typically exhibit higher levels of self-efficacy and self-worth, consistent with findings from Quaye et al. (2024). This positive psychological state enables individuals to mobilize cognitive and emotional resources more effectively and to maintain positive self-evaluations when facing adversity, challenges, or stress. Thus, a clear self-identity provides a psychological resource reservoir that enhances the ability to recover from setbacks and demonstrates heightened mental toughness. Taken together, the chain mediation model proposed in this study outlines a clearer intervention pathway for enhancing mental toughness. It not only emphasizes the independent mediating roles of social support and self-identity but also underscores their sequential mediating relationship, offering deeper insights into the complex influence of team dynamics on psychological well-being.

## 7 Conclusion and implications

### 7.1 Conclusion

The study concludes that: (1) Team cohesion significantly and positively influences athletes’ mental toughness. Both task cohesion and relationship cohesion contribute to the formation of a positive, harmonious, and stable team atmosphere, which in turn provides athletes with a strong psychological foundation for coping with setbacks and adversity. (2) Social support and self-identity each exert partial mediating effects on the relationship between team cohesion and mental toughness, with social support demonstrating a stronger mediating effect than self-identity. (3) Social support and self-identity also exhibit a chain mediating relationship, wherein team cohesion enhances the perception of social support, which subsequently strengthens self-identity, ultimately leading to increased mental toughness. These findings clarify the multi-level mechanisms through which team dynamics influence athletes’ psychological adaptation. Additionally, this study sees team cohesion and social support as conditional resources, and self-identity as a personality trait resource. These two kinds of resources work together to shape athletes’ mental toughness. This view helps explain how mental toughness develops and also adds to the COR theory.

### 7.2 Implications

1. This study defines team cohesion and social support as important resources in COR theory. Traditional COR theory looks more at support from individual resources. This study focuses on how team cohesion, as a larger social resource, affects people through its unique way of building resources. In practice, improving team cohesion and creating a positive team atmosphere should be main strategies to boost athletes’ mental toughness. Coaches and managers can do this by planning team-building activities and increasing cooperation and trust among team members.
2. Build a social support network with many parts. Team managers should set up a support system that includes care from coaches and help from teammates. They should hold regular team meetings, watch athletes’ emotions, and give help when needed. At the same time, they should build a strong mental health support network so athletes get enough help when facing challenges.
3. Prioritize the development of athletes’ self-identity. Coaches, teammates, and administrators should acknowledge individual contributions and assign meaningful team roles to help athletes internalize a sense of identity and value within the group.
4. Implement integrated intervention strategies to cultivate mental toughness. In line with the chain mediation model proposed in this study, resilience development should not depend on a single factor but should adopt a combined model of “cohesion–support–identity.” This approach unites external resource inputs—such as team cohesion and social support—with the internal psychological process of self-identity formation to produce a more robust and sustainable effect. This model not only validates the mediating effect but also provides a detailed depiction of complex psychological mechanisms, offering a paradigm for research on multiple mediations and chain effects.

## 8 Limitations

Firstly, due to the reliance on questionnaire-based methods, this study was unable to adequately capture the long-term effects of team cohesion on athletes’ mental toughness or establish a clear causal relationship. Future research may employ longitudinal surveys or experimental designs to explore causal pathways and dynamic changes among variables more thoroughly. Secondly, this study primarily examined the mediating roles of social support and self-identity, excluding other potentially influential mediators. Incorporating additional mediating variables in future research could offer a more comprehensive understanding of the mechanisms by which team cohesion influences mental toughness. Finally, regarding sample selection, the sample was limited to Chinese athletes, which may restrict the generalizability of the findings across different cultural contexts and occupational groups. Future research should aim to expand the diversity of the sample to include participants from various regions and backgrounds, enabling cross-group comparative studies to test the robustness and applicability of the findings.

## Data Availability

The raw data supporting the conclusions of this article will be made available by the authors, without undue reservation.
